# Self-Regulatory Responses to Unattainable Goals: The Role of Goal Motives

**DOI:** 10.1080/15298868.2014.889033

**Published:** 2014-02-25

**Authors:** Nikos Ntoumanis, Laura C. Healy, Constantine Sedikides, Alison L. Smith, Joan L. Duda

**Affiliations:** 1 College of Life and Environmental Sciences, University of Birmingham, Birmingham B15 2TT, UK; 2 School of Psychology, University of Southampton, Shackleton Building (B44), Highfield Campus, Southampton SO17 1BJ, UK; 3 School for Education, University of Bath, Bath, Claverton, Down Bath, North East Somerset BA2 7AY, UK

**Keywords:** Goal pursuit, Goal regulation, Disengagement, Reengagement, Goal motives

## Abstract

Does motivation for goal pursuit predict how individuals will respond when confronted with unattainable goals? Two studies examined the role of autonomous and controlled motives when pursuing an unattainable goal without (Study 1) or with (Study 2) the opportunity to reengage in alternative goal pursuit. Autonomous motives positively predicted the cognitive ease of reengagement with an alternative goal when the current goal was perceived as unattainable, especially when participants realized goal unattainability relatively early during goal striving. Autonomous motives, however, were negative predictors of cognitive ease of disengagement from an unattainable goal. When faced with failure, autonomously motivated individuals are better off realizing early the goal unattainability. Otherwise, they will find it difficult to disengage cognitively from the pursued goal (despite reengaging cognitively in an alternative goal), possibly due to interfering rumination.

Hollywood movies often depict characters who determinedly and heroically confront challenges and live happily thereafter. Daily life is quite different for common mortals. Individuals need to be strategic in how they invest or re-allocate their limited resources in pursuit of important goals. A great deal of research has examined how goals are activated (Fishbach & Ferguson, 2007), planned (Gollwitzer & Oettingen, 2011), or monitored (Zimmerman & Paulsen, 1995) to produce successful performance. Comparatively less is known about persistence in the face of increasing difficulty or about strategic goal disengagement (Shah, 2005). Reactions to goal-related challenges have implications, not only for future goal striving and performance, but also for psychological well-being (Sheldon & Elliot, 1999; Smith, Ntoumanis, Duda, & Vansteenkiste, 2011). The objective of the reported research is to examine (a) how individuals respond when confronted with unattainable goals and (b) the role of motivation for goal pursuit in predicting such responses.

Issues related to the pursuit of challenging goals, goal re-investment, and futile persistence are highly relevant in achievement contexts. Sport is an ideal domain to study such issues, because it is achievement-driven: goal-setting and goal-regulation are endemic in sport settings, and perceptions of success are enhanced by evidence of triumph over mounting adversity (Weinberg, Burke, & Jackson, 1997). It is for these reasons that we focused on sport as the context of our investigation.

## The “Why” of Goal Pursuit

Our main interest is to find out how the motives underlying personal goals can explain variations in self-regulatory responses to goal challenges. Conceptually, our work draws from the self-concordance model (Sheldon & Elliot, 1999) which is concerned with the “why” of goal striving. Rooted in Self-Determination Theory (SDT; Deci & Ryan, 1985, 2002), this model proposes that the motives underlying goal striving can be differentiated according to their level of internalization within the self, and advocates the benefits of high self-concordant or autonomous motives (e.g., enjoyment, personal importance) as opposed to low self-concordant or controlled motives (e.g., guilt, social approval) for goal striving. Autonomous motives reflect greater integration of goals into the self and, thus, greater alignment with relatively enduring personal interests and core values. Consequently, autonomous motives have greater volitional strength and result in higher goal-directed effort and goal attainment than controlled motives. Even when goals are assigned to individuals by an external source (e.g., a coach), as it is often the case in achievement settings, they can be internalized within the self and striven for with autonomous motives. Evidence has documented the adaptive role of autonomous goal motivation across a variety of life strivings, ages, and cultures (Sheldon, Houser-Marko, & Kasser, 2006; Sheldon & Kasser, 1995; Sheldon, Ryan, Deci, & Kasser, 2004). In sport, cross-sectional and season-long studies have shown that autonomous motives are related positively to goal effort and attainment as well as increased psychological well-being; in contrast, controlled motives are unrelated to goal effort or attainment, but are directly and negatively related to psychological well-being (Smith, Ntoumanis, & Duda, 2007; Smith et al., 2011).

Research on the “why” of goal striving has been relatively restrictive in its scope, focusing primarily on the positive implications of autonomous goal striving. Although the advantages of such striving in mobilizing and allocating resources have been well-documented (Ntoumanis et al., in press; Sheldon, 2008), goal pursuit is rarely without its challenges. The obstacles encountered during goal striving and the ways in which individuals cope with such obstacles are crucial considerations that lack adequate empirical attention (Brandstätter & Schüler, 2013). The present research makes the case for the importance of examining the motivational predictors and behavioral consequences of strategic disengagement and reengagement in an alternative goal, when attainment of the original goal becomes highly unlikely.

## Goal Disengagement and Alternative Goal Engagement

In certain situations, personal goals become no longer attainable. Anticipated gains are threatened, and the decision to abandon personal goals signals failure in reaching desired objectives (Carver & Scheier, 1990). The reasons that cause a goal to become unattainable vary as a function of the nature of the goal and the longevity of goal striving. At the level of life goals (Schmuck & Sheldon, 2001), changes in physical and biological capabilities, as well as progression through life transitions, can present obstacles to goal attainment. At the level of daily personal strivings, competing tasks and time constraints can prevent goal fulfillment. In sport, daily and more long-term goals can become unattainable through injury, biological limitations, or other constraining factors (e.g., superior play by an opponent).

Wrosch, Scheier, Carver, and Schulz (2003) proposed that the negative consequences associated with unattainable goals can be alleviated through effective goal disengagement. Such disengagement prevents the accumulation of failure experiences and frees personal resources for future goal striving. These authors provided evidence for the positive associations of goal disengagement with psychological well-being and physical health. Moreover, Wrosch, Miller, Scheier, and Pontet (2007) highlighted the further self-regulatory benefits of alternative goal engagement, also labeled goal reengagement. This involves one or more of the following: identify new paths to the same goals, identify new goals that serve the same higher-order goal or develop new goals altogether (Carver & Scheier, 2005). Goal reengagement is purported to ease the distress caused by unattainable goals by providing future opportunities for goal attainment (Wrosch et al., 2003, 2007). Wrosch and colleagues maintained that, despite their mutual benefits, goal disengagement and alternative reengagement are relatively independent self-regulatory processes. For example, disengaging from an unattainable goal may not necessarily lead to alternative reengagement.

Goal disengagement and full reengagement in alternative important goals may therefore provide a route through which the negative consequences of goal unattainability are negotiated effectively. However, letting go of cherished goals and moving on to pursue alternative goals might be easier said than done. Wrosch et al. (2003, 2007) maintained that individuals vary greatly in their ability to reduce effort and commitment towards their personal goals. To date, such inter-individual variability in goal disengagement and alternative reengagement situational responses has received scant empirical attention. Wrosch et al. (2003) suggested that self and personality processes contribute to the implementation of these two self-regulatory strategies. In the current research, we considered the role of goal motives as predictors of goal disengagement and alternative reengagement.

What is the relation between goal motives and individuals’ responses to unattainable goals? We discuss several plausible links and exploratory hypotheses based on the properties of such motives, as per the self-concordance model literature. With regard to goal disengagement, autonomous motives reflect a greater level of ownership over one's goal striving (Sheldon & Elliot, 1999). Thus, this perceived freedom may enable individuals to disengage when they consider their goals unattainable. In contrast, the internal and external pressures accompanying striving with controlled motives may prevent individuals from freely disengaging, even when efforts appear futile. For example, a desired external reward that depends upon the attainment of a sports goal may dissuade an athlete from disengaging fully, even when physical constraints prevent the goal from realization. Although these hypotheses are theoretically plausible, the links between goal motives and goal disengagement can be viewed from a different angle. The greater personal investment and commitment in autonomously pursued goals, which are more closely aligned with the self, combined with the typically greater relative progress made toward such goals during goal striving (Koestner, Otis, Powers, Pelletier, & Gagnon, 2008), may render it more difficult to disengage from these goals. In contrast, disengagement may provide an opportunity to free oneself from the pressures of controlled motives, and hence a positive relation was expected between controlled goal motives and ease of disengagement.

With regard to alternative goal engagement, striving toward a new goal requires additional motivational strength and resources. On the basis of the demonstrated motivational benefits of autonomous motives for goal-directed effort (Sheldon & Elliot, 1999), it is likely that these motives will facilitate the identification and initiation of a new goal in the face of failure. In contrast, controlled motives are unlikely to lead to alternative goal engagement as they have been found to be unrelated to goal progress and persistence (Koestner et al., 2008).

## Overview

The aim of this research was to test whether the motivation that underpins goal striving can predict self-regulatory responses to goal challenges in achievement-focused settings. Answers to this previously unaddressed research question can reveal synergies between the self-determination and self-regulation literatures. We conducted two studies in controlled settings that manipulated goal difficulty. In the second study, we examined mediators and behavioral outcomes of the relation between goal motives and goal regulation, which were the key independent and dependent variables, respectively, in both studies.

In Study 1, we manipulated feedback to ensure that all participants were faced with an unattainable personal goal. Participants had the opportunity to disengage or persist, but they did not have the opportunity to reengage in a different goal. We gathered measures of personal goal motives and ease of cognitive disengagement/alternative goal engagement. Study 2 was a variation of Study 1 in that participants were offered the opportunity to engage in an alternative goal pursuit that served the same higher level goal (Carver & Scheier, 2005). We measured not only the cognitive ease of disengagement and reengagement, but also the behavioral choices of disengagement without reengagement (i.e., quitting), disengagement with reengagement, and futile persistence with the original goal. Further, in Study 2, we identified when the goal was perceived as clearly unattainable and tested whether the timing of this realization impacted on disengagement and reengagement responses (both cognitive and behavioral). Finally, we examined whether the effects of personal goal motives on these responses were indirect via rumination and via shame or embarrassment. In both studies, our central hypothesis was that autonomous personal goal motives would predict self-regulatory responses adaptive to the experimental situation (i.e., disengagement, followed by reengagement). However, in each study, we addressed unique but complementary research questions.

## Study 1: Responses to a “Real” Unattainable Goal

We measured autonomous and controlled motives as well as cognitive ease of disengagement and reengagement in the presence of an unattainable goal. We used a laboratory setting to examine goal regulation responses while participants experienced actual goal failure. We offered no hypotheses as to how autonomous goal motives would predict ease of disengagement, since, as explained earlier, arguments could be made for considering such motives as positive predictors and negative predictors of goal disengagement. In contrast, we predicted that controlled motives will be positively linked to the ease of disengagement. With regard to ease of alternative goal engagement, we hypothesized that autonomous motives would be positive predictors of reengagement, whereas controlled motives would be unrelated to reengagement.

### Method

#### Participants

We recruited 66 athletes (44 male, 24 female; *Mage* = 20.52, *SDage* = 1.35) from the University of Birmingham. The participants were regularly training (*M* = 2.98 hours per week, *SD* = 1.31) athletes from a variety of team and individual sports (e.g., rugby and tennis).

#### Procedure

Participants completed the study individually in a single laboratory session. On arrival, they were fitted with a heart rate monitor, and they filled out consent forms and a demographics questionnaire. Participants learned that they were taking part in a study concerned with experiences of repeated goal striving on a cycle-ergometer. The procedure involved a warm-up, an incremental submaximal exercise test, a 2-minute baseline trial, and an 8-minute goal trial on a cycle ergometer. The purpose of the submaximal test was to establish the workload required to perform at 50% of predicted maximum heart rate (220 heart beats per minute minus age), when working at 100 revolutions per minute. The workload on the ergometer was fixed at this level for the subsequent cycle trials to standardize the impact of exercise intensity on participants’ psychological responses (Ekkekakis, 2003). For the 2-minute cycle trial, participants were asked to cover the greatest possible distance. The purpose of this test was to calculate personal goals for the subsequent trials. Upon completion of the 2-minute trial, participants were informed of their personal distance goals for the subsequent 8-minute goal trial and were told that successful attainment of their goal would enable progression to the 10-minute goal trial. In reality, no participant attempted the 10-minute trial due to “failure” in the 8-minute trial.

In the 8-minute trial, participants pursued a goal that was based on the distance they covered during the 2-minute baseline trial. This goal was calculated by multiplying the 2-minute distance by 4, and then further multiplying by 95%. Pilot testing confirmed that this procedure created perceptions of a difficult yet plausible goal prior to the 8-minute trial. During the 8-minute trial, participants were constantly reminded of their goal and were provided with feedback regarding the time elapsed, distance covered, distance remaining to attain the goal, as well as percentage of the distance covered. This information was updated every minute. During the trial, the distance feedback was manipulated by computer software to make the goal appear unattainable. Extensive pilot work verified the plausibility of the manipulated feedback and a post-trial checking confirmed this for all participants. At the end of the task, we solicited participants’ open-ended written feedback on the study, which was actually a suspicion probe. No participant expressed suspicion regarding the manipulated feedback. We debriefed participants via email once testing had been completed.

### Measures

#### Goal-Related Variables for the Cycling Task

Prior to the 8-minute goal trial, participants rated their personal goal motives and goal attainment expectancies. Specifically, participants rated (1 = *not at all*, 7 = *very much so*) the extent to which they were pursuing their goal for extrinsic (“because you feel you are expected to do so”), introjected (“because you would feel embarrassed or anxious if you didn't”), identified (“because the goal will give you personally important information”), or intrinsic (“because of the enjoyment or challenge the pursuit of the goal provides you”) reasons. Aligned with self-concordance model research (Sheldon et al., 2004), we created an autonomous motives score by averaging the scores for the identified and intrinsic motivation items. Likewise, we formed a controlled motives score from the mean response to the extrinsic and introjected items. Participants also responded to four items (adapted from Sheldon & Elliot, 1999; e.g., “How confident are you that you will achieve your goal?”) related to their goal attainment expectancies (1 = *not at all*, 7 = *very much so*). Following the trial, participants rated their cognitive ease of disengagement/reengagement. They responded to 10 items (1 = *strongly disagree*, 5 = *strongly agree*) adapted from Wrosch et al. (2003). Four items pertained to goal disengagement (e.g., “It was easy for me to stop thinking about my cycling goal and let it go”) and six items pertained to goal reengagement (e.g., “I put effort towards other meaningful goals in the cycling task”).

### Results

#### Descriptive Statistics, Scale Reliabilities, and Correlations

We display the descriptive statistics, scale reliabilities, and correlations for the variables used in the hypothesized model in Table 1.

**TABLE 1:** Descriptive Statistics, Internal Reliabilities, and Correlations among Study 1 Variables

	*M*	*SD*	*α*	1	2	3
1. Autonomous motives	4.62	1.28	.78	–		
2. Controlled motives	4.01	1.33	.67	−.22	–	
3. Disengagement	2.33	.83	.89	−.42**	.06	–
4. Reengagement	3.14	1.04	.94	.34**	.16	.11

Note: * *p* < .05, ** *p* < .01.

#### Goal Motives, Goal Disengagement, Goal Reengagement, and Enjoyment

We ran two multiple regression analyses with autonomous and controlled motives predicting simultaneously ease of reengagement and disengagement. The first regression showed that autonomous motives (*β* = .36, *p* < .01), but not controlled motives (*β* = .20; *p* = .10), predicted ease of reengagement (*R*^2^ = .16). The second regression showed that autonomous motives (*β* = –.42, *p* < .01), but not controlled motives (*β* = .01, *p* = .96), predicted ease of disengagement (*R*^2^ = .18). When we reran the model controlling for gender, goal attainment expectancies for the 8-minute trial, and hours of sport training per week, the aforementioned regression coefficients remained largely unchanged.

### Discussion

We addressed a novel question: how does goal motivation predict the cognitive ease of disengagement and reengagement, when faced with an unattainable goal? We obtained a potentially interesting and conceptually plausible pattern of results with regard to autonomous goal motivation. The results indicated that autonomous goal motivation was negatively related to the cognitive ease of disengagement. This finding could be explained by the fact that individuals invest more psychological and physical resources to autonomously pursued goals, because such goals are more closely aligned with the self (Sheldon & Elliot, 1999). Further, progress towards goal attainment is typically greater when motivation for goal striving is autonomous (Koestner et al., 2008). Thus, abandoning such goals, even in the face of highly probable future failure, is not easy. However, autonomous motives were also positively related to the ease of alternative goal engagement. Hence, although finding difficult to “abandon” mentally the unfulfilled goal, individuals with high autonomous goal motivation do not simply give up, but instead start making reengagement plans. In contrast, controlled motives were unrelated both to disengagement (which was unexpected) and reengagement (which was expected). Given that self-worth is “on the line” when motivation is controlled (Deci & Ryan, 2002), disengagement might provide an opportunity to free oneself from the pressures and contingencies that controlled motives represent. Alternative goal engagement also carries the risk of failure and, as demonstrated previously (Smith et al., 2011), controlled motivation is typically not associated with behavioral investment in the face of adversity. Taken together, Study 1 indicates that controlled motivation for goal pursuit does not result in adaptive self-regulatory responses, when the pursued goal becomes unattainable.

Study 1 had two limitations that could compromise the generalizability of its findings. To begin, participants could have reengaged by setting a new distance goal. According to Carver and Scheier's (2005) model, reengagement could involve scaling back the original lower-order goal. It is likely that some participants in our study could have scaled back the distance-related goal for the 8-minute trial by setting a new goal for that trial (unfortunately, we did not include behavioral measures of persistence). The reengagement items capture this possibility. We did not explicitly provide such alternative goals to participants, but in many achievement domains individuals are likely to scale back their goal when the original goal is no longer attainable (Lench & Levine, 2008). Attaining the reduced-distance goal could have given these participants a modicum of accomplishment, despite not having met their original goal. Regardless, it is useful for a follow-up study to test another possibility (based on Carver & Scheier's model) for goal reengagement. Here, participants would be offered a higher-order goal and, when the lower-order goal would no longer be attainable, they would have the option to disengage behaviorally from the unattainable goal and reengage in a new goal that also serves the higher-order goal. Study 1 has another limitation. In a manipulation check, all participants confirmed post-trial that their goal was unattainable, but we did not ask them during the 8-minute trial to specify the exact time point at which they felt their goal was unattainable. Reengagement opportunities via different means of goal engagement and the time point at which one realizes that a goal is unattainable could be vital in determining variability in cognitive and behavioral disengagement and reengagement. We addressed both limitations of Study 1 in our follow-up study.

## Study 2: Responses to a “Real” Unattainable Goal when Behavioral Reengagement is Available

Similar to the previous study, in Study 2 we measured autonomous and controlled motives as well as the cognitive ease of disengagement and reengagement in the face of an unattainable goal. Further, in Study 2 we measured three behavioral responses to an unattainable goal: (futile) persistence, disengagement without reengagement, and disengagement with reengagement. Unlike Study 1, participants in Study 2 were given the option to disengage after failure and reengage behaviorally in an alternative task pursuing a goal that served the same higher-level goal. According to Carver and Scheier (2005), one adaptive self-regulatory response to an unattainable goal is to disengage and reengage in an alternative goal serving the same higher-level goal. We used a stronger failure feedback manipulation in Study 2 in order to facilitate the earlier realization that the pursued goal was unattainable and to give participants a greater opportunity to reengage. We reasoned that, the earlier in the trial participants realized that their goal was unattainable, the more likely they would be to reengage in the alternative task.

### 

#### Goal Motives and Self-Regulation of Goals

We hypothesized that goal motives would relate to the cognitive ease of reengagement and disengagement in the same manner as in Study 1. In terms of behavioral responses to failure, we reasoned that they might follow the same pattern as the cognitive ones. Thus, we expected those who persisted and those who disengaged and reengaged to be more autonomous in their motivation, and we expected those who disengaged without reengaging to be more controlled in their motivation.

#### The Intermediary Role of Shame/Embarrassment and Rumination

We went a step further from Study 1 in testing processes that potentially explain the difficulty (or not) of individuals with autonomous and controlled motives to disengage cognitively when faced with unattainable goals. Specifically, we examined rumination and shame/embarrassment following the realization that the initially pursued goal is no longer attainable. Ruminative thinking is instigated when goal progress is unsatisfactory or under threat (Koole, Smeets, Van Knippenberg, & Dijksterhuis, 1999). Also, perseverance of goal-related thoughts following failure occurs with goals that are central to one's identity (Brunstein & Gollwitzer, 1996; Martin & Tesser, 1989); such goals, according to the self-concordance model (Sheldon & Elliot, 1999), are pursued with autonomous motivation. Thus, it is possible that autonomous goal motivation is negatively linked to cognitive ease of disengagement (as shown in Study 1), because individuals ruminate about failure of central goals (Brandstätter & Schüler, 2013). Thus, we hypothesized autonomous motives to be linked positively with rumination, and the latter to predict the ease of disengagement (negatively).

In terms of controlled motives, we reasoned that the pressures perceived when striving with controlled motives are likely to evoke shame and embarrassment when realizing goal failure (Deci & Ryan, 2008). Disengagement might be a way out of this negative affective state. Thus, we hypothesized that controlled motives would predict positively feelings of shame/embarrassment and that the latter would predict positively cognitive disengagement.

#### When Realizing that the Pursued Goal is Unattainable

We also examined whether time of realization of the unattainability of the pursued goal would influence thoughts about and behavioral actions reflecting disengagement and reengagement. We hypothesized that those who realized relatively early (at least half-way through the trial) that their goal was unattainable would find it easier to disengage and reengage (cognitively and behaviorally) than those who realized the goal unattainability much later in the trial. In the former case, participants would perceive that there was sufficient time to accomplish successfully the higher-order goal by reengaging in another goal that served that higher-order goal. Finally, we explored the links between goal motives, shame/embarrassment, rumination, and self-regulation responses in the subsample of participants who realized relatively early the unattainability of the pursued goal. We speculated that, in this subsample, autonomous goal motivation would be more strongly related to cognitive and behavioral reengagement than in the whole sample.

#### Goal Motives and Behavioral Investment

Further, we examined the relation between goal motives and objective behavioral investment (i.e., actual distance covered and heart rate measures) for participants who (a) persisted in the cycling trial after realizing that their goal was unattainable and (b) reengaged in cycling. Based on the self-concordance literature (Koestner et al., 2008), we hypothesized that, in both cases, behavioral investment would be positively predicted by autonomous motives and would be unrelated to controlled motives.

### Method

#### Participants

We recruited 86 regularly training athletes (53 men, 33 women; *Mage* = 20.78 years, *SDage* = 3.35) from the University of Birmingham in exchange for course credit or financial reward (£5). These athletes were from a variety of team sports and trained on average for 3.83 hours per week (*SD* = 2.35). We excluded from the analysis those who did not realize that their goal was unattainable until the end of the trial (*n* = 2) or had a missing value for this question and a behavioral response of futile persistence (*n* = 4). In essence, this was a manipulation check to exclude those participants who did not perceive their goal as unattainable. Due to the nature of the task, a pre-trial manipulation check of goal unattainability would have been nonsensical. We also excluded one male participant due to expressed suspicion that the feedback was manipulated. The final sample comprised 79 participants.

#### Procedure

The procedure was a variation of Study 1. There was one key difference, in that we gave participants in Study 2 the choice to reengage in an alternative goal on a rowing ergometer if they felt their goal was unattainable during the 8-minute trial. Participants learned that the study was part of a national fitness survey taking place in universities across the UK. Participants were also told that if they were successful in achieving their distance goal (either on the cycle or rowing ergometer), their information would be added to a national database; as such, a higher-level goal was created (Carver & Scheier, 2005) that was suggested to be attainable via either fitness equipment. For feedback, in addition to the information shown to participants as in Study 1 (e.g., time elapsed, distance covered), a value displayed the (fictitious) expected percentage that participants should have attained if they were on track to achieve their goal. This value was ostensibly based on the average percentage reached by previous participants who had achieved their goal. Thus, the failure-focused feedback manipulation was more potent than in Study 1, so that participants would be more likely to feel that the goal was unattainable early in the trial and that reengagement was a realistic alternative toward the achievement of the higher level goal before experiencing too much fatigue. When participants felt that the goal was unattainable, they had the option to (a) persist (futilely) in the cycling, (b) disengage from the goal, or (c) disengage from the cycling goal and reengage in an alternative goal on a rowing ergometer.

If participants chose to reengage, they had an alternative goal to achieve in the remaining time. For example, if they completed 4 minutes of the cycling trial, they had a goal to achieve in the 4 remaining minutes on the rowing ergometer. The distance for the rowing goal was determined using information from an earlier 2-minute baseline trial on the rowing ergometer. We did not manipulate performance feedback in the rowing task. Prior to engaging in that alternative task, participants completed a few questionnaires (see below). This allowed them to rest before engaging in the rowing goal, and also ensured that their responses related to their experiences of the cycling trial (i.e., responding to an unattainable goal). Testing was complete when participants had either finished the cycling task (by cycling for the full 8 minutes or disengaging) or had completed the rowing task, and had responded to all post-trial measures. No participant disengaged from the rowing task. The procedures for suspicion checking and for debriefing were identical to those of Study 1.

#### Goal-Related Variables for the Cycling Task

Prior to the cycling trial, we measured goal motives (as in Study 1). We also asked participants to respond to three goal difficulty items (e.g., “how difficult is your goal?”) and three goal efficacy items (e.g., “how strong is your belief that you are able to achieve your goal?”) that we developed for the purposes of this study (1 = *not at all*, 7 = *very much so*). We measured goal difficulty and efficacy, because these variables affect goal striving (Locke & Latham, 2002). Further, at the end of the cycling trial, similar to Study 1, participants rated items relating to their cognitive ease of disengagement from the cycling task and reengagement with the rowing task, using measures that we adapted from Wrosch et al. (2003). We also asked participants at every minute of the trial to report their goal attainment expectancies. At the end of the cycling trial, they reported at which minute they felt their goal was unattainable (by reviewing their minute-by-minute goal attainment expectancy scores).

#### Fear of Experiencing Shame and Embarrassment

At the end of the trial (as it was not possible for participants to complete questionnaires while cycling at high speed), participants rated (1 = *not at all*, 7 = *very much so*) the extent to which they felt embarrassment and shame when they realized during the cycling trial that they could not achieve their goal. We used the Fear of Experiencing Shame and Embarrassment scale of the Performance Failure Anxiety Index (Conroy, Willow, & Metzler, 2002). This scale has seven items (e.g., “Having failed to attain the goal in the trial I feel that everybody will know I've failed”).

#### Rumination

At the end of the trial, we assessed retrospectively participants’ rumination after they realized during the cycling trial that they could not achieve their goal. Participants responded to five items (1 = *not at all*, 5 = *very much so*) following the cycling trial. We adapted those items from the Rumination on Sadness Scale (Conway, Csank, Holm, & Blake, 2000). The items asked participants to rate the extent to which they were focusing on their failure to achieve the target goal (e.g., “I had difficulty getting myself to stop thinking about my failure”).

#### Additional Variables

We also recorded heart rate pre-trial, at every minute during the cycling trial and, when applicable, at every minute during the rowing trial.

### Results

#### Preliminary Analyses

We present descriptive statistics, scale reliabilities, and correlations in Table 2. We assessed differences in cognitive disengagement and reengagement between the three behavioral choices [futile persistence (*n* = 37)/disengagement (*n* = 14)/disengagement with reengagement (*n* = 28)] with a one-way Multivariate Analysis of Variance (MANOVA). This produced significant differences between the groups (Wilks' Λ = .34, *F* (4, 150) = 26.97, *p* < .001, partial η^2^ = .42), with significant univariate effects for both the ease of cognitive disengagement (*F* (2, 79) = 6.22, *p* = .003, partial η^2^ = .14) and reengagement (*F* (2, 79) = 58.67, *p* < .001, partial η^2^ = .61). Follow-up Bonferroni comparisons revealed that participants who persisted with the cycling task had lower scores for ease of disengagement than participants who disengaged (*p* = .003). Furthermore, participants who chose to reengage in the rowing task had higher scores for cognitive reengagement than those who persisted or disengaged without reengaging (*p* < .001).

**TABLE 2:** Descriptive Statistics, Internal Reliabilities, and Correlations among Study 2 Variables

	*M*	*SD*	*α*	1	2	3	4	5
Whole sample (*N* = 79)								
1. Autonomous motives	4.43	1.34	.59	–				
2. Controlled motives	4.35	1.34	.66	.16	–			
3. Shame/embarrassment	2.54	1.19	.88	.05	.49**	–		
4. Rumination	2.38	.87	.81	.41**	.23*	.60**	–	
5. Disengagement	2.65	.94	.68	−.24*	−.16	−.27*	−.44**	–
6. Reengagement	2.20	1.36	.97	.18	−.07	.18	.11	.10
Subsample (*n* = 54)								
1. Autonomous motives	4.39	1.32	.71	–				
2. Controlled motives	4.29	1.39	.65	.17	–			
3. Shame/embarrassment	2.55	1.06	.83	.01	.47**	–		
4. Rumination	2.31	.93	.93	.51**	.26	.62**	–	
5. Disengagement	2.89	.90	.63	−.28*	−.16	−.41*	−.49**	–
6. Reengagement	2.33	1.40	.96	.21	−.16	−.04	.07	.04

*Note*: * *p* < .05, ** *p* < .01. The subsample includes participants who realized early that the goal was unattainable.

#### Goal Motives, Goal Disengagement, and Goal Reengagement

We tested the relations between autonomous and controlled motives, as well as the cognitive ease of goal disengagement and goal reengagement, in an effort to test the replicability of the pattern of relations among these variables obtained in Study 1. Consistent with Study 1, multiple regression analysis showed that autonomous motives (*β* = –.25, *p* = .02), but not controlled motives (*β* = .11, *p* = .30), predicted ease of disengagement (*R*^2^ = .08). Further, consistent with Study 1, controlled motives (*β* = –.11, *p* = .32) did not predict ease of reengagement, but, unlike Study 1, autonomous motives (*β* = .16, *p* = .15) also did not predict reengagement (*R*^2^ = .03). The results remained largely unchanged when we controlled for gender, hours of sport training per week, goal difficulty, and goal efficacy. In order to assess differences in autonomous and controlled motives between the three behavioral choices, we conducted a one-way MANOVA. No significant differences in the levels of goal motives across the behavioral choices emerged: Wilks’ Λ = .96, F (4, 150) = .72, *p* = .58, and partial η^2^ = .02.

#### The Role of Shame/Embarrassment and Rumination

We subsequently tested the indirect effects of goal motives on goal disengagement via rumination and shame/embarrassment. We used Hayes' (2009) Process macro which provides unstandardized regression coefficients for direct and indirect effects as well as their 95% bias corrected bootstrap confidence intervals. If the confidence intervals of the indirect effects do not include zero, then there is evidence of mediation. We tested for indirect effects even if there was no significant relation between the independent and the dependent variables (see Hayes, 2009). In summary, the results showed that autonomous motivation predicted rumination (*b* = .26, *p* < .01), which in turn predicted ease of disengagement (*b* = –.43, *p* < .01); the indirect effect (*b* = –.11) did not encompass zero (95% bias corrected confidence interval = –.20 to –.05). Controlled motivation predicted embarrassment (*b* = .47, *p* < .01), embarrassment was predictive of ease of disengagement (*b* = –.19, *p* = .04), and the indirect effect did not encompass zero (*b* = –.09, 95% bias corrected confidence interval = –.19 to –.01).

In terms of behavioral choices, a one-way MANOVA showed no significant differences in the levels of rumination and shame/embarrassment: Wilks’ Λ = .97, *F* (4, 150) = .64, *p* = .63, partial η^2^ = .02.

#### The Moment One Realizes that the Goal is Unattainable: Time Matters

We tested whether the time when participants realized that the goal was unattainable resulted in differences in cognitive disengagement and reengagement experienced. We reran the regressions including only those participants (*n* = 54) who realized that their goal was unattainable by half-way through the trial (the median for this variable was the 4th minute). The results, compared to those obtained from the whole sample, evinced similar non-significant paths from controlled motives to both reengagement (*β* = –.20, *p* = .15) and disengagement (*β* = –.11, *p* = .41). Although the path from autonomous motives to disengagement was similar in strength (but no longer significant; *β* = –.26, *p* = .06), the path from autonomous motives to reengagement was substantially larger and marginal (*β* = .24, *p* = .08). When comparing behavioral responses, however, we obtained no significant differences in the levels of goal motives among the futile persistence, disengagement without reengagement, and disengagement with reengagement groups: Wilks’ Λ = .93, *F*(4, 100) = .97, *p* = .43, partial η^2^ = .04.

Next, we compared participants who felt that the goal was unattainable up to the 5th minute (*n* = 54) with participants who felt the goal was unattainable at or after the 5th minute (*n* = 25). We first conducted a one-way MANOVA with cognitive disengagement and reengagement as the dependent variables. A significant effect emerged (Wilks’ Λ = .86, *F* (2, 76) = 6.11, *p* = .003, partial η^2^ = .14). For ease of disengagement, those who felt that the goal was unattainable earlier found it easier to disengage than those who perceived the goal to be attainable for longer, *F*(1, 79) = 11.44, *p* = .001, partial η^2^ = .13. There were no differences in ease of reengagement, *F*(1, 77) = 1.42, *p* = .24,

606

partial η^2^ = .02. In terms of differences in behavioral choices between the two groups, a chi-square analysis indicated that participants were more likely to disengage or reengage, if they perceived that the goal was unattainable before than after the 5th minute, χ^2^(2) = 10.18, *p* = .006.

Goal Motives and Behavioral Investment Exerted

We conducted additional analyses to examine further the relation between goal motives and behavioral investment. In the whole sample, autonomous motivation was linked to higher persistence, as indicated by this type of motivation marginally predicting later realization in the trial of goal unattainability (and by implication greater distance covered; autonomous motives *β* = .21, *p* = .07; controlled motives *β* = .009, *p* = .94), as well as predicting higher percentage of maximum heart rate (maxHR) prior to realizing the goal was unattainable (autonomous *β* = .36, *p* < .001; controlled *β* = –.08, *p* = .41). For participants who persisted in the cycling task (*n* = 37), autonomous motives predicted a greater percentage of distance being covered per minute after the goal was perceived to be unattainable (*β* = .47, *p* = .005), compared to controlled motives (*β* = –.13, *p* = .39). With regard to the heart rate measures, for those who persisted in the cycling trial, autonomous motives predicted positively (*β* = .19, *p* = .05) and controlled motives predicted negatively (*β* = –.20, *p* = .03) the percentage of maximum heart rate (maxHR) achieved after the goal was perceived to be unattainable, even when controlling for participants’ resting HR and the percentage of maxHR achieved prior to the goal being perceived as unattainable. When examining the percentage of rowing distance covered by those who chose to reengage (*n* = 28), neither autonomous motives (*β* = –.22, *p* = .28) nor controlled motives (*β* = .30, *p* = .14) were significant predictors. The small size of the subsample may have contributed to the null findings. Neither goal motive predicted the percentage of maxHR achieved for those who reengaged with the rowing task (autonomous *β* = –.08, *p* = .75, controlled *β* = .13, *p* = .56).

### Discussion

Study 2 built upon and extended Study 1 by offering participants the opportunity to disengage from the pursued unattainable goal and to reengage behaviorally in an alternative goal serving the same higher-level goal. Unlike Study 1, we measured both cognitive and behavioral aspects of disengagement and reengagement. Preliminary analyses showed that the two facets of self-regulatory responses to goal failure were related. In terms of cognitive disengagement and reengagement, their pattern of relations with autonomous and controlled motives was somewhat consistent with the pattern of relations found in Study 1. Autonomous motives were negatively related to ease of disengagement and positively related to ease of reengagement, although the latter effect was marginal and was observed for those participants who realized early the unattainability of the pursued goal. In contrast, controlled motives were unrelated to disengagement and reengagement. Interestingly, for those who chose to persist in cycling, autonomous motives predicted higher, objectively assessed, behavioral investment. Although this last finding is at face value consistent with the self-concordance model literature (Sheldon & Elliot, 1999; Smith et al., 2007, 2011), the pursued goal in the case of the cycling task was unattainable; thus persistence was futile and counter-productive to the attainment of the higher level goal.

We also examined the intermediary role of rumination and shame/embarrassment, following the realization of goal unattainability, in linking goal motives with disengagement responses. In terms of the cognitive aspects of these self-regulation responses, those with autonomous motives found it more difficult to disengage cognitively from an unattainable goal, partly because they ruminated more. This finding aligns with past evidence indicating that perseverance of goal-related thoughts following failure is more likely, when goals are central to one's identity (Brunstein & Gollwitzer, 1996; Martin & Tesser, 1989). Shame and embarrassment were predicted by controlled motives. This finding was in accordance with our hypothesis that the pressures perceived when striving with controlled motives would elicit shame and embarrassment upon realization of goal failure (Deci & Ryan, 2008). Further, this negative affective state predicted negatively the ease of cognitive disengagement. This finding was unexpected, as we hypothesized a positive relation between the two variables; in other words, disengagement would represent a way out of the embarrassment associated with failure. It is possible that the competitive nature of the task and the public display of performance meant that, even when ashamed, participants did not find it easy to disengage. It is worth exploring whether, with less competence-based tasks or normative evaluation settings, the relation between these two variables would be positive. The indirect effect of controlled motives on ease of disengagement was significant, despite the lack of direct effect. Hence, the results indicate that the relation between controlled motivation and goal disengagement might be more complex and indirect (which might explain the non-significant finding in Study 1) compared to the autonomous motivation–goal disengagement link.

Additionally, we examined whether the timing of realization of the unattainability of pursued goal would influence thoughts and behavioral options about disengagement and reengagement. We hypothesized that those who realized goal unattainability relatively early would find it easier to disengage and reengage cognitively and behaviorally than those who realized goal unattainability later on in the trial. We obtained partial support for this hypothesis. Participants who perceived their goal as unattainable early found it easier to disengage (cognitively and behaviorally) and reengage (behaviorally) than those who took longer to come to the same realization. Further, in the former group of participants, the positive effect of autonomous motives on cognitive reengagement increased substantially in size and became marginal. Thus, realization of goal unattainability earlier during the goal striving process may be beneficial in terms of adaptive self-regulation. However, it is not always easy to determine whether difficulties during the goal striving process can be overcome with greater effort and persistence (as in previous self-concordance literature) or should be interpreted as advanced warnings of impending goal failure and should lead to strategic disengagement and re-allocation of goal striving efforts to preserve one's resources. Future research will do well to examine whether factors such as previous task experience, mindfulness, and support or advice from the immediate social environment can help individuals to decide whether to “hold” or “fold” their goals.

Although autonomous motives were influential in predicting cognitive responses to goal unattainability, they did not discriminate among the three behavioral choices (but note that autonomous motives predicted higher behavioral investment in participants who chose to persist). The categorical nature of the behavioral choices variable and the relatively small cell sizes, especially for disengagement without reengagement, could have contributed to the null findings. However, it is also possible that behavioral and other types of responses to goal unattainability are partly determined by contextual motivational factors, such as the extent to which significant others are autonomy supportive or controlling (Mageau & Vallerand, 2003). A limitation of our research was that it restricted examination of motivational factors to personal goal motives. Future investigations could consider creating autonomous and controlling motivational environments (either via explicit instructions or priming procedures; Hodgins, Yacko, & Gottlieb, 2006) and examine the influence of situational motivation, as well as the interaction between personal goal motivation and situational motivational, on persistence, goal flexibility, strategic disengagement, and affective reactions to goal unattainability.

### General Discussion

Perseverance in the pursuit of an important goal has often been glorified in ancient and modern cultures. In contrast, “giving up” is frequently taken as an indication of weakness and lack of determination. While effort and commitment are key pre-requisites for goal attainment (Locke & Latham, 2002), there are situations where persistence is futile. In such situations, the adaptive self-regulatory response for maintaining psychological well-being and health is disengagement. This involves the abandoning the pursued goal and reengaging in an alternative important goal (Shah, 2005; Wrosch et al., 2003, 2007). In the reported studies, we relied on the self-concordance model (Sheldon & Elliot, 1999) to examine how autonomous and controlled motivation for goal striving (the two key independent variables) predict goal disengagement and alternative goal engagement responses (the two key dependent variables), when striving for unattainable goals. This was the primary research question we posed. We also examined mediators and outcomes of the relations between goal motives and goal regulation. We controlled for and showed that the effects of goal motives were not reducible to goal-related variables that are known to affect goal pursuit, such as goal attainment expectancies, goal difficulty, and goal efficacy. The findings clarify conceptual ambiguities as to why and how autonomous motives are associated with goal flexibility or rigidness in the face of increased goal difficulty. Also, the findings make a unique contribution by identifying an interesting link between the self-determination and self-regulation literatures.

#### Goal Motivation and Self-Regulation of Goal Strivings

The results pertaining to autonomous motivation were mixed. Autonomous goal motivation positively predicted the cognitive ease of alternative goal engagement in the case of goal unattainability, albeit in Study 2 this effect was marginal and only for participants who realized early the goal unattainability. In both studies, autonomous goal motivation was also associated with difficulties to disengage cognitively from the unattainable goal. These difficulties were partly due to rumination and resulted in high levels of persistence with the unattainable goal when not behaviorally disengaging (Study 2). These results can be explained with reference to the functional perspective on goal disengagement (Jostmann & Koole, 2009). According to this perspective, explicit goals can remain activated in working memory for extended time periods. In the case of unattainable goals, sustained goal activation is problematic, because it results in rumination, uncontrollable thoughts, and unwanted feelings.

With regard to controlled motives, the absence of any major predictive effects on goal regulation responses is aligned with previous self-concordance research that has highlighted the lack of contribution of these motives to the goal striving process (Koestner et al., 2008; Smith et al., 2007, 2011). The other noteworthy result was that these motives predicted shame/embarrassment after realizing that the pursued goal was unattainable. Taken together, the findings indicate that controlled motivation for goal pursuit will not result in adaptive self-regulation responses, when the pursued goal is difficult or unattainable. There are situations, however, in which controlled motivation may lead to reengagement and short-lived persistence; such situations entail cases when participants are ego-involved (Ryan, Koestner, & Deci et al., 1991). In Study 2, among participants who reengaged, the path coefficient between controlled motives and persistence (i.e., distance covered) was *β* = .30. Although non-significant, probably due to the small sample size, this effect indicates that there are circumstances where controlled motives may contribute to goal striving. However, longitudinal studies have provided extensive evidence demonstrating that controlled motives are unrelated to sustained effort, goal progress, and attainment (Koestner et al., 2008; Smith et al., 2007, 2011) and can be detrimental for psychological well-being (Miquelon & Vallerand, 2008).

#### Conclusions, Limitations, and Future Research Directions

Although there is extensive conceptual and empirical evidence to support the beneficial role of holding autonomous motives for goal striving and success, our results indicate that individuals with high autonomous motives find it difficult to disengage cognitively from such goals when they become unattainable. This issue could be addressed by helping these individuals to realize earlier the futility of their goal striving. Also, the timing of this realization does matter, cognitively and behaviorally. Helping individuals develop relevant experience and thus enabling them to form more accurate assessments of goal difficulty, facilitating awareness of internal and external limits, and providing a variety of alternatives for goal reengagement are some of the ways in which an earlier realization of goal unattainability can be accomplished. Jostmann and Koole (2009) also suggest that opening up one's working memory to external information will result in unattainable goals being revised, replaced, or relinquished. This memorial opening-up can be achieved via facilitating perceptual information about the external world and accessing one's implicit motives.

Some limitations of our work may be addressed by future research. While we assessed in both studies how much participants valued their assigned goal (as reflected in their responses to the identified regulation question that assessed autonomous motivation), we did not measure how much participants valued the alternative goal that we offered in Study 2 (but we told participants that the alternative goal served the same higher order goal). Follow-up research will need to measure motivation for the new goal striving and could entail offering several choices in terms of both original and alternative goal engagement. Choice is one of the defining features of an autonomy-supportive contextual environment, which fosters autonomous motivation (Deci & Ryan, 2002). Follow-up research could also consider the assessment of implicit motives for goal striving. Incongruence between explicit and implicit motivation can act as a “hidden stressor” and impact on subjective well-being and health (Baumann, Kaschel, & Kuhl, 2005). In addition, future research could examine multiple facets of disengagement. Jostmann and Koole (2009) discussed not only cognitive and behavioral disengagement, but also affective disengagement (i.e., down-regulating unwanted feelings) and motivational disengagement (i.e., rejecting explicit goals which are not in line with one's implicit motives). Potentially, similar distinctions could be made in terms of reengagement. Understanding the multiple facets of disengagement/reengagement and their potential interplay will enable researchers to predict better responses to goal difficulties as well as the contributing role of motivational factors. The consequences of unfulfilled goals on subsequent goal pursuit also invite empirical attention. Failing to achieve a goal can produce poor performance on subsequent tasks that require executive function (Masicampo & Baumeister, 2011). This finding regarding goal failure may have implications for movement efficiency in subsequent task execution—a critical performance factor in many sports (e.g., golf). Future cross-domain investigations of goal striving would also be useful, as adaptive and maladaptive self-regulation responses to goal difficulties (e.g., in sport) may interfere with subsequent goal pursuit in other life domains (e.g., school).

Carver and Scheier (2005) remarked that “for successful negation of the challenges life provides, we believe yet another kind of competence is also important: the ability to know when to continue the effort to reach a goal, and when to disengage and let it go” (p. 543). Our findings provide an endorsement for these remarks. The findings, in addition, highlight the relevance of the motivation underlying goal striving in painting a broader picture of this competence.
